# Implementing Mobile Health–Enabled Integrated Care for Complex Chronic Patients: Patients and Professionals’ Acceptability Study

**DOI:** 10.2196/22136

**Published:** 2020-11-20

**Authors:** Jordi de Batlle, Mireia Massip, Eloisa Vargiu, Nuria Nadal, Araceli Fuentes, Marta Ortega Bravo, Jordi Colomina, Reis Drudis, Montserrat Torra, Francesc Pallisó, Felip Miralles, Ferran Barbé, Gerard Torres, CONNECARE-Lleida Group

**Affiliations:** 1 Group of Translational Research in Respiratory Medicine Institut de Recerca Biomedica de Lleida Lleida Spain; 2 Center for Biomedical Network Research in Respiratory Diseases Madrid Spain; 3 eHealth Unit Eurecat, Centre Tecnòlogic de Catalunya Barcelona Spain; 4 Gerència Territorial de Barcelona Institut Català de la Salut Barcelona Spain; 5 Atenció Primària Àmbit Lleida Lleida Spain; 6 Research Support Unit Lleida Fundació Institut Universitari per a la recerca a l'Atenció Primària de Salut Jordi Gol i Gurina Lleida Spain; 7 Centre d'Atenció Primària Cappont Gerència Territorial de Lleida Institut Català de la Salut Lleida Spain; 8 Universitat de Lleida Lleida Spain; 9 Servei de Cirurgia Ortopèdica i Traumatologia Hospital de Santa Maria de Lleida Lleida Spain; 10 Unitat de Dolor Agut Hospital de Santa Maria de Lleida Lleida Spain; 11 Institut de Recerca Biomedica de Lleida Lleida Spain

**Keywords:** mHealth, eHealth, patient acceptance of health care, patient satisfaction, health plan implementation, chronic diseases

## Abstract

**Background:**

Integrated care (IC) can promote health and social care efficiency through prioritization of preventive patient-centered models and defragmentation of care and collaboration across health tiers, and mobile health (mHealth) can be the cornerstone allowing for the adoption of IC.

**Objective:**

This study aims to assess the acceptability, usability, and satisfaction of an mHealth-enabled IC model for complex chronic patients in both patients and health professionals.

**Methods:**

As part of the CONNECARE Horizon 2020 project, a prospective, pragmatic, 2-arm, parallel, hybrid effectiveness-implementation trial was conducted from July 2018 to August 2019 in a rural region of Catalonia, Spain. Home-dwelling patients 55 years and older with chronic conditions and a history of hospitalizations for chronic obstructive pulmonary disease or heart failure (use case [UC] 1), or a scheduled major elective hip or knee arthroplasty (UC2) were recruited. During the 3 months, patients experienced an mHealth-enabled IC model, including a self-management app for patients, a set of integrated sensors, and a web-based platform connecting professionals from different settings or usual care. The Person-Centered Coordinated Care Experience Questionnaire (P3CEQ) and the Nijmegen Continuity Questionnaire (NCQ) assessed person-centeredness and continuity of care. Acceptability was assessed for IC arm patients and staff with the Net Promoter Score (NPS) and the System Usability Scale (SUS).

**Results:**

The analyses included 77 IC patients, 58 controls who completed the follow-up, and 30 health care professionals. The mean age was 78 (SD 9) years in both study arms. Perception of patient-centeredness was similarly high in both arms (usual care: mean P3CEQ score 16.1, SD 3.3; IC: mean P3CEQ score 16.3, SD 2.4). IC patients reported better continuity of care than controls (usual care: mean NCQ score 3.7, SD 0.9; IC: mean NCQ score 4.0, SD 1; *P*=.04). The scores for patient acceptability (UC1: NPS +67%; UC2: NPS +45%) and usability (UC1: mean SUS score 79, SD 14; UC2: mean SUS score 68, SD 24) were outstanding. Professionals’ acceptability was low (UC1: NPS −25%; UC2: NPS −35%), whereas usability was average (UC1: mean SUS score 63, SD 20; UC2: mean SUS score 62, SD 19). The actual use of technology was high; 77% (58/75) of patients reported physical activity for at least 60 days, and the ratio of times reported over times prescribed for other sensors ranged from 37% for oxygen saturation to 67% for weight.

**Conclusions:**

The mHealth-enabled IC model showed outstanding results from the patients’ perspective in 2 different UCs but lacked maturity and integration with legacy systems to be fully accepted by professionals. This paper provides useful lessons learned through the development and assessment process and may be of use to organizations willing to develop or implement mHealth-enabled IC for older adults.

## Introduction

In recent decades, socioeconomic development has increased life expectancy and led to progressively aging populations with an increased burden of chronic diseases [1]. This increased disease burden has heavily affected the already overburdened health and social care systems, which struggle to provide adequate services with limited resources. Traditional care models are falling short in adequately responding to the needs of chronic patients. The struggle of different care settings to communicate efficiently among themselves leads to care fragmentation and patients being left with the feeling of starting anew after every transition [2]. Moreover, patients are often passive actors without the necessary empowerment and no clear role in their own management [2]. Overall, there is a need for a profound redesign of how care is provided to older people with chronic health conditions, with a focus on patient participation, care quality, and system sustainability [3]. Integrated care (IC) models were created to address these challenges, with the aim of generating efficiencies through the adoption of patient-centered models, promotion of efficient continuity of care across settings, and prioritization of preventive strategies [4]. eHealth and mobile health (mHealth) can be the cornerstone allowing for the adoption of IC models [5].

In this scenario, the Horizon 2020 European Union Research and Innovation project CONNECARE—Personalized Connected Care for Complex Chronic Patients—attempted to co-design, develop, deploy, and evaluate a smart adaptive IC model for complex chronic patients (CCPs) [6]. From April 2016 to December 2019, participants in CONNECARE co-designed and experienced an organizational model supported by an eHealth platform that allows IC. The IC model allowed shifting from a conventional reactive care to a home-based preventive model built on cross-setting collaboration and interoperability, patient empowerment, and health risk prediction and management based on the analysis of patient-specific and population-based data. The model was supported by an advanced eHealth platform, based on information and communication technologies and Internet of Things, offering a cross-setting web-based Smart Adaptive Case Management (SACM) system for professionals and an mHealth self-management app with 3-level monitoring for patients. Finally, the CONNECARE IC model and supporting eHealth platform were tailored to different settings involving CCPs across Europe, including Lleida and Barcelona in Spain, Groningen in the Netherlands, and Ashdod in Israel.

Most novel IC interventions are assessed in light of the Triple Aim compass, which enhances patient experience, improves population health, and reduces overall costs [7]. According to the Triple Aim concept, patients’ acceptability and satisfaction are key aspects for the large-scale adoption of novel management strategies. However, it is equally important that the involved health and social care professionals feel that any change in their routines will allow them to provide better care to their patients. This key concept has been described well in the Quadruple Aim, which expands Triple Aim to include the improvement of the work life of clinicians and staff [8]. Therefore, the assessment of acceptability and satisfaction in patients and professionals should be an unavoidable aspect in the evaluation of novel IC strategies, complementing effectiveness and cost-effectiveness assessments.

The CONNECARE mHealth-enabled IC model is being deployed and tested in different European settings. This paper focuses on the assessment of acceptability and satisfaction in patients and professionals in relation to the implementation of mHealth-enabled IC in the rural region of Lleida—Catalonia (Spain)—which had historically struggled with low levels of cross-setting interoperability, limiting the capacity of professionals from different settings to provide a coordinated response to patients’ needs, and limited patient empowerment, with patients having mostly passive roles throughout their care paths.

## Methods

### Study Design

A prospective, pragmatic, 2-arm, parallel, type 1 hybrid effectiveness-implementation trial [9] that assesses patients and professionals’ acceptability of a 3-month mHealth-enabled IC intervention as compared with that of usual care was conducted. The study was conducted from July 2018 to August 2019 in Lleida, which is a large rural area of more than 4300 km^2^, including 2 tertiary hospitals, University Hospital Arnau de Vilanova and University Hospital Santa Maria, and a network of 23 primary care centers spread across the whole territory, providing services to 400,000 citizens.

### Target Population

The intervention was deployed for 2 different use cases (UCs): (1) home-dwelling patients, with chronic conditions and a history of visits to the emergency room (ER) leading to hospitalizations (UC1); and (2) home-dwelling patients, with chronic conditions, undergoing a major elective hip or knee arthroplasty surgery (UC2). The specific eligibility criteria included the following: age more than 55 years, having a hospital admission because of a respiratory or cardiovascular event (UC1), having a programmed major elective hip or knee arthroplasty surgery (UC2), not having dementia or cognitive impairment (Global Deterioration Scale<5 [10]), LACE index for readmission score>7 (UC1) [11], American Society of Anesthesiologists Physical Status Classification System II or III (UC2) [12], being assigned to a primary care center in the region, living at home and being discharged back to the community, and passing a basic technological test assessing home connectivity and patients and/or care givers’ competence with the use of technology (Multimedia Appendix 1 [13,14]).

### Recruitment

Patients were recruited either during an unanticipated admission to the hospital through the ER (UC1) or at the time of surgery (UC2). All patients were identified based on data from electronic medical records (EMRs) and contacted by a case manager. Regardless of the UC, once a patient was recruited for the intervention arm, the search for a similar control began. All patients and their caregivers, regardless of the UC and study arm, received a face-to-face explanation about the study.

### Intervention

Patients in the intervention arm experienced an mHealth-enabled IC model, including (1) a preliminary assessment of the patient’s health status using several questionnaires, tests, and indices specific to their main chronic diseases and social needs done before hospital discharge for UC1 patients and at the time of scheduled surgery for UC2 patients; (2) access to a self-management app with status and performance reports, a virtual coach with customizable automated feedback, and full communication with the care team and guidance on its day-to-day use, taking into account that the app could be managed directly by the patients or indirectly by the informal caregivers or relatives; (3) a Fitbit Flex 2 (Fitbit) digital activity tracker [15] and additional sensors deemed necessary by the care team [16]: digital pulse-oximeter, digital scale, and digital blood pressure monitor, all of them fully integrated into the self-management app; (4) a patient’s profile in the SACM web-based platform, which would be accessible by all the involved professionals (hospital, primary, and social care) and used to coordinate and communicate with professionals in the different settings, control the patient’s evolution, and contact the patient if needed; and (5) assignment of a case manager in charge of supervising the whole process and being the main patient contact point. Additional details on the IC model and the required implementation efforts can be found in Multimedia Appendix 1. It must be noted that, as part of the CONNECARE project, the supporting technology used by patients and professionals was being developed and fine-tuned throughout the study. Usual care arm patients were managed from primary care.

### Data Collection

Patients’ characteristics were collected at recruitment, including age, sex, main chronic diseases, Charlson Comorbidity Index [17], Barthel Index for Activities of Daily Living [18], and the Pfeiffer Mental Status Questionnaire [19]. The main patient outcomes were collected after 3 months and included patient’s perception of person-centeredness, assessed by the Person-Centered Coordinated Care Experience Questionnaire (P3CEQ) [20]; patient’s perception of continuity of care, assessed by questions G1 to G5 of the Nijmegen Continuity Questionnaire (NCQ) [21]; satisfaction with the IC platform in IC arm patients and staff, assessed by the Net Promoter Score (NPS) [22] and the System Usability Scale (SUS) [23]; and the actual use of the different elements of the IC platform by patients. The NPS was based on the question “How likely is it that you would recommend our system CONNECARE to a family member or friend?” to be answered in a 0 (*not at all likely*) to 10 (*extremely likely*) scale. Individuals scoring 9 or 10 were considered as *promoters*, individuals scoring 7 or 8 as *passives*, and individuals scoring 0 to 6 as *detractors*. The final NPS score was obtained by subtracting the proportion of *detractors* from the proportion of *promoters*, and it could range from −100% to +100% (a positive score is considered good, +50% is considered excellent, and anything more than +70% is exceptional) [24].

### Statistical Analyses

Participants’ baseline characteristics were described by the number (percentage), mean (SD), or median (P25-P75), as appropriate. Comparisons between IC and usual care patients’ baseline characteristics were performed using chi-square test, *t* test, or Kruskal-Wallis test, as appropriate. Comparisons between IC and usual care patients’ person-centeredness and continuity of care were performed using chi-square test or *t* test, as appropriate, excluding patients answering “Don’t know” or “No answer.” Satisfaction with the IC platform in IC patients and staff were described using mean (SD) or median (P25-P75), as appropriate. Finally, the actual use by patients of the different elements of the IC platform was described by reporting the proportion of times reported over times prescribed, using median (P25-P75). Data analyses were conducted using Stata, version 12.1 (StataCorp). The threshold for significance was set at .05.

### Implementation Framework

The Consolidated Framework for Implementation Research (CFIR) [13] was used to assess implementation aspects. A detailed description of the implementation strategies and framework can be found in Multimedia Appendix 1.

### Ethical Considerations

This study was approved by the Ethics Committee of Hospital Arnau de Vilanova (CEIC-1685), and all participants provided written informed consent. All collected data were handled and stored in accordance with current National and International legislation.

## Results

**Figure 1:**
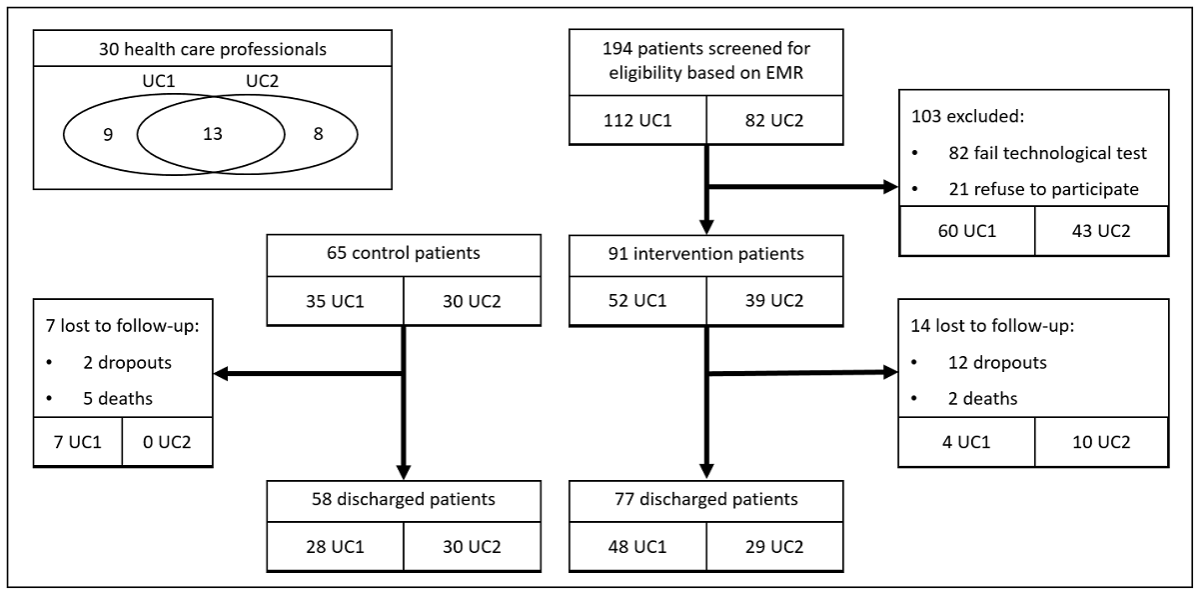
Study flowchart. EMR: electronic medical record; UC: use case.

In this study, up to 194 patients were screened for eligibility (112 for UC1 and 82 for UC2). After excluding patients who did not meet the inclusion criteria, 91 patients were recruited for the intervention arm and 65 for the usual care arm. Final analyses were based on 77 IC patients and 58 usual care control patients who completed the follow-up and 30 health care professionals (Figure 1).

The main characteristics of the patients included in the study are shown in Table 1. The mean age was 78 (SD 9) years in both study arms, with patients in UC1 being 10 years older than patients in UC2 on average. Patients in UC1 had a higher burden of comorbidities than patients in UC2 (Charlson Comorbidity Index in UC1: mean 6.9, SD 2.1; UC2: mean 4.2, SD 1.6; *P*<.001). No statistically significant differences were found between patients in the usual care arm and patients in the IC arm, regardless of UC. Regarding the analyses of health care professionals, UC1 included 1 hospital case manager, 3 hospital physicians, 3 primary care case managers, 9 primary care physicians, and 6 primary care nurses, and UC2 included 1 hospital case manager, 1 hospital physician, 1 hospital surgeon, 1 hospital anesthesiologist, 2 primary care case managers, 9 primary care physicians, and 6 primary care nurses.

**Table 1:** Baseline characteristics of patients in usual care and integrated care.

Characteristics	Use case 1				Use case 2		
	Usual care (n=28)	Integrated care (n=48)	*P*^a^ value	Usual care (n=30)		Integrated care (n=29)	*P*^a^ value
Sex (male), n (%)	17 (61)	24 (50)	.37	8 (27)		12 (41)	.23
Age (years), mean (SD)	82 (8)	82 (7)	.88	73 (8)		72 (9)	.50
Charlson^b^ (score), mean (SD)	7.4 (2.1)	6.7 (2.0)	.15	4.2 (1.7)		4.2 (1.5)	.88
Barthel^c^ (score), median (P25-P75)	90 (73-95)	90 (68-100)	.40	95 (90-100)		100 (95-100)	.16
Pfeiffer^d^, NI^e^, n (%)	21 (75)	37 (77)	.67	30 (100)		27 (93)	.14

^a^Chi-square test, *t* test, or Kruskal-Wallis equality-of-populations rank test, as appropriate.

^b^Charlson Comorbidity Index.

^c^Barthel Index for Activities of Daily Living.

^d^Pfeiffer Mental Status Questionnaire.

^e^NI: no impairment.

Regardless of UC and intervention arm, the perception of patient-centeredness was very high, with a mean P3CEQ score of 16.1 (SD 3.3) in the usual care arm and 16.3 (SD 2.4) in the IC arm. Regarding the continuity of care, patients in IC scored better than patients in usual care (usual care: mean NCQ G1-G5 score 3.7, SD 0.9; integrated care: mean NCQ G1-G5 score 4.0, SD 1.0; *P*=.04). Further information on patient-perceived person-centeredness and continuity of care can be found in Multimedia Appendix 1.

Patients and professionals’ satisfaction with the deployed technology for IC is presented in Tables 2 and 3. Patients in UC1 reported an overall NPS score of +67% and patients in UC2 of +45%, whereas professionals involved in UC1 scored −25% and in UC2 −35%. Similarly, although the mean score for SUS was 75 (SD 19), the mean score for professionals was 61 (SD 20).

**Table 2:** Integrated care patients’ satisfaction with the technology.

Measures		All (n=77)	UC^a^1 (n=48)	UC2 (n=29)
**NPS^b^ questions (0 [poor] to 10 [good]), median (P25-P75)**				
	Overall satisfaction	10 (8-10)	10 (8-10)	10 (8-10)
	Easiness of use	9 (8-10)	9 (8-10)	8 (5-10)
	Ability to be used without help	9 (7-10)	9 (8-10)	8 (5-10)
	Would you recommend it	10 (8-10)	10 (8-10)	9 (8-10)
NPS score (−100% to +100%)		+58%	+67%	+45%
**SUS^c^ score (0 [awful] to 100 [excellent])**				
	Mean (SD)	75 (19)	79 (14)	68 (24)
	Users scoring over 68 points, n (%)	54 (70)	38 (79)	16 (55)

^a^UC: use case.

^b^NPS: Net Promoter Score.

^c^SUS: System Usability Scale.

**Table 3:** Staff’s satisfaction with the technology.

Measures		All (n=30)	UC^a^1 (n=22)	UC2 (n=21)
**NPS^b^ questions (0 [poor] to 10 [good]), median (P25-P75)**				
	Overall satisfaction	6 (5-8)	6 (5-8.5)	6.5 (5-8)
	Easiness of use	6 (6-7)	6.5 (5-8)	6 (4.5-7.5)
	Ability to be used without help	6 (5-9)	6.5 (5-9)	6 (5.5-9)
	Would you recommend it	6.5 (5-8)	6.5 (5.5-8.5)	6.5 (5-7.5)
NPS score (−100% to +100%):		−29%	−25%	−35%
**SUS^c^ score (0 [awful] to 100 [excellent])**				
	Mean (SD)	61 (20)	63 (20)	62 (19)
	Users scoring over 68 points, n (%)	14 (47)	10 (46)	9 (43)

^a^UC: use case.

^b^NPS: Net Promoter Score.

^c^SUS: System Usability Scale.

Table 4 reports on the actual use of technology by the patients experiencing IC. The actual use of the physical activity tracker was outstanding, with up to 77% (58/75) of patients having reported measures over 60 days out of 90. The ratio of times reported over times prescribed for the rest of sensors that could be proposed to patients ranged from 37% for oxygen saturation to 67% for weight. Finally, the use of the messaging function allowing patients to ask or answer requirements for or from the professional care team was high, with a median (P25-P75) of 19 (10.5-41) in UC1 and 10 (5-22) in UC2 over the 90-day intervention.

Finally, the results of the evaluation of the implementation according to CFIR can be found in Multimedia Appendix 1.

**Table 4:** Integrated care patients’ use of technology.

Technologies		All (n=77)	UC^a^1 (n=48)	UC2 (n=29)
**Daily steps (Fitbit)**				
	Days reported, median (P25-P75)	86 (63-92)	86 (63-92)	87 (68.5-92)
	Users reporting ≥60 days, n (%)	58 (77)	36 (77)	22 (79)
**Weight (Withings), median (P25-P75)**				
	Times R/P^b^	67 (42-91)	67 (42-91)	NU^c^
**Blood pressure (Withings), median (P25-P75)**				
	Times R/P	41 (32-50)	43 (37-50)	38 (21-58)
**Heart rate (Withings), median (P25-P75)**				
	Times R/P	42 (36-51)	42 (36-51)	NU
**Oxygen saturation (SpO_2_; Withings), median (P25-P75)**				
	Times R/P	37 (22-42)	37 (22-42)	NU
**Body temperature (Withings), median (P25-P75)**				
	Times R/P	41 (36-48)	NU	41 (36-48)
**Messages to the care team, median (P25-P75)**				
	Total number	18 (7-37)	19 (10.5-41)	10 (5-22)

^a^UC: use case.

^b^R/P: reported or prescribed.

^c^NU: not used in the UC.

## Discussion

### Principal Findings

The assessment of the patients and professionals’ acceptability of an mHealth-enabled IC program targeting home-dwelling CCP patients with a history of hospitalizations (UC1) or undergoing a major elective hip or knee arthroplasty surgery (UC2) showed 2 different perceptions. Although patients and/or informal caregivers or relatives reported a very high acceptability of the IC program and its supporting technology, professionals rated it as moderately poor. Patients reported very high perceptions of patient-centeredness, continuity of care, satisfaction, and usability of the IC platform, and matching with these positive perceptions, actual use of the different features of the IC platform was high. Although patients positively qualified their user experience [25], professionals felt the burden of a system under constant development, which in turn limited their experience and translated to moderately poor satisfaction with the IC platform.

### Strengths and Limitations

This study has several strengths: (1) from day 1, an effort was made to involve all actors from different organizations who would participate in a large-scale deployment of the mHealth-supported IC program, which is key as the lack of cooperation between organizations and professionals is a well-known barrier for the implementation of IC [4]; (2) the involvement of informal carers as actors being important in facilitating the use of the patient’s app in older patients; (3) the prescription and monitoring of patients’ physical activity, as mobility impairment is frequent in people older than 65 years [26]; and (4) the geography of the implementation area, a large rural region of more than 4300 km^2^, which could benefit the most from community-based integrated care initiatives that precluded unnecessary visits to primary care centers or hospitals. Similarly, there were several limitations: (1) the IC platform was in a constant process of refinement and addition of new functionalities; thus, the user experience was richer by the end of the implementation study compared with the very beginning; and (2) having a single entry point to the IC program, which was the hospital either after an ER admission (UC1) or at the time of surgery (UC2), as it is important that system-wide cross-organizational care pathways consider multiple entry points [27]. However, primary care centers are currently being considered as a potential additional entry point if the IC system is further implemented in the region.

### Patients’ Perspective: Comparison With Previous Work

The use of mHealth apps for patients with chronic conditions has been explored in the last decade, showing the potential for appropriate security level, effective monitoring, self-management, and communication [28]. However, a 2016 review of Apps for Heart Failure symptom monitoring and self-care reported that a minority of the available apps had the required quality, content, or functionality [29]. Our IC platform aimed to go beyond a patient’s app and established a comprehensive IC model, including a patient’s app, a portfolio of different sensors linked with the app, and a professional’s web-based platform for monitoring, communication with the patient, and collaboration among professionals in different health settings. The first indicator of the success of an mHealth intervention is the rate of dropouts. In our study, only 13% of patients in the IC arm decided to abandon the program. For instance, Bentley et al [30] reported that half of the participants in an mHealth-based self-management intervention for chronic obstructive pulmonary disease (COPD) withdrew from the study. The scores for acceptability (UC1: NPS +67%; UC2: NPS +45%) and usability (UC1: mean SUS score 79, SD 14; UC2: mean SUS score 68, SD 24) were outstanding. These results are better than most results obtained in existing mHealth tools targeting chronic patients, for example, mHealth tools aiming to improve quality of life in breast cancer survivors such as BENECA (NPS +7%) [31] or Oncokompas (NPS −36%) [32], an Intelligent Virtual Assistant for promoting behavior change and self-care in older people with type 2 diabetes (mean SUS 74, SD 13) [33], or a gait-monitoring mobile phone app for older users (mean SUS 60, SD 11) [34]. Similarly, interventions targeting surgical patients by means of education and communication apps have obtained positive results in terms of acceptability and usability [35,36], comparable with results obtained with automated phone messaging platforms [37,38]. Finally, preliminary results of variants of the CONNECARE IC model implemented in other European settings have been positive. For instance, the CONNECARE self-management app was rated positively by older patients with cancer who were offered remote home monitoring after surgery (mean SUS 74, SD 19; NPS +29%) [39].

### Patients’ Perspective: Lessons Learned

The key factors for the success of our comprehensive IC model from the patients’ perspective have been (1) including a comprehensive set of features with the patient’s app acting as a hub of services including the integration of monitoring devices, in line with a 2016 review of Apps for Heart Failure symptom monitoring and self-care reporting that a minority of the available apps had the required content or functionality [29]; (2) the involvement of patients since early phases of development, as proposed by Lundell et al [40] in a recent qualitative analysis of the use of home telemonitoring in patients with COPD; (3) the flexibility of potential end users, as the app could be managed directly by the patients (most UC2 patients) or by a relative or informal carer (most UC1 patients); (4) enabled bidirectional communication with the care team, potentially avoiding unnecessary visits to primary care centers or hospital; (5) appropriate feedback on the daily monitoring and patients’ achieved goals, including personalized motivational advice; (6) push-up notifications to remind key events, tasks, or goals; and (7) ease of use and quality-of-life features, such as being translated to the different official languages in the region (Catalan and Spanish) or having several display settings including font size, as difficulty in using the technology is a common reason for withdrawal [30].

### Professionals’ Perspective

According to the Quadruple Aim, the improvement of the work life of clinicians and staff is a key factor for the adoption of new health programs [8]. In this study, the scores for professionals’ acceptability were low (UC1: NPS −25%; UC2: NPS −35%), whereas scores for usability were moderately high (UC1: mean SUS score 63, SD 20; UC2: mean SUS score 62, SD 19). However, these ratings were directly related to the temporal constraints of the study setting, as professionals were required to use a system in a dynamic development and implementation process rather than a fully developed one. However, having the opportunity to directly participate in the development of the IC model and platform allowed professionals to feel engaged and propose changes and new features to be developed, which ultimately resulted in great engagement (no professionals dropped out of the implementation study). Understanding the factors influencing professionals’ adoption of eHealth is complex [41], but the ability to provide quality care is key [42]. On the one hand, the potential of the IC platform to provide quality care was the key to professionals’ engagement; being able to monitor key aspects of chronic diseases or monitoring pain after surgery, enabling communication between carers in different care settings; having the option to prescribe and monitor physical activity; or case managers having access to a geographical representation of patients in a map, with the possibility of selecting patients based on predefined characteristics or generating optimized routes for home visits, showed professionals the game-changing features of the platform. On the other hand, a system under constant development, not achieving a full integration with legacy EMR systems and the coexistence of 2 management systems (usual care and IC) at the same time (which implied some duplicity of tasks) were the main barriers to adoption.

### Challenges for Large-Scale Deployment

Although usability and acceptability are key for the adoption of mHealth-enabled IC, large-scale adoption requires cost-effectiveness and an adequate reimbursement and payment model. On the one hand, regarding cost-effectiveness, the implementation of our IC model reduced unplanned contacts with the health system, reduced health costs, and was cost-effective, as reported elsewhere. On the other hand, designing of an imbursement and payment model capable of accommodating the costs of new roles and required technologies, while fully benefiting from the savings in terms of reductions in the use of health and social care resources, can be challenging, especially when the model involves different organizations and providers. Therefore, a firm positioning of the involved health authorities and governing bodies is required to fully fulfill the ambition of our mHealth-enabled IC model. The use of the CFIR framework highlighted the key barriers and facilitators for large-scale adoption (Multimedia Appendix 1).

### Conclusions

The assessment of the patients and professionals’ acceptability of an mHealth-enabled IC program showed outstanding results from the patients’ perspective. However, the web-based professionals’ platform needs to be fully matured and fully integrated into legacy systems before moving forward toward large-scale deployment. This paper, thus, provides useful lessons learned through the development and assessment process and may be of use for organizations willing to develop or implement mHealth-enabled IC for older adults.

## Appendix

Multimedia Appendix 1 Implementing mobile health–enabled integrated care for complex chronic patients: a patients and professionals’ acceptability study.

## Abbreviations

CCP: complex chronic patient
CFIR: Consolidated Framework for Implementation Research
COPD: chronic obstructive pulmonary disease
EMR: electronic medical record
ER: emergency room
IC: integrated care
mHealth: mobile health
NCQ: Nijmegen Continuity Questionnaire
NPS: Net Promoter Score
P3CEQ: Person-Centered Coordinated Care Experience Questionnaire
SACM: Smart Adaptive Case Management
SUS: System Usability Scale
UC: use case
