# Supplementary material for: Implementing Mobile Health–Enabled Integrated Care for Complex Chronic Patients: Patients and Professionals’ Acceptability Study
Source: JMIR Mhealth Uhealth. 2020 Nov 20;8(11):e22136. doi: 10.2196/22136 (PMC7718089; doi:10.2196/22136)
Supplement: Multimedia Appendix 1 [file mhealth_v8i11e22136_app1.docx]

**SUPPLEMENTAL INFORMATION**

Implementing mHealth-enabled Integrated Care for Complex Chronic Patients: a Patients and Professionals’ Acceptability study

Jordi de Batlle, PhD, Mireia Massip, BSc(Nurs), Eloisa Vargiu, PhD, Nuria Nadal, MD, Araceli Fuentes, MD, Marta Ortega Bravo, MD, Jordi Colomina, MD, Reis Drudis, MD, Montserrat Torra Riera, MD, Francesc Pallisó, PhD, Felip Miralles, PhD, Ferran Barbé, MD, Gerard Torres, MD, on behalf of the CONNECARE-Lleida group.

Contents

[Basic technological test for patients and carers 2](#_Toc49428038)

[The Integrated Care model 2](#_Toc49428039)

[Technical structure of the supporting eHealth platform 8](#_Toc49428040)

[Implementation efforts 9](#_Toc49428041)

[Implementation framework 9](#_Toc49428042)

[References 10](#_Toc49428043)

[Supplementary tables 11](#_Toc49428044)

# Basic technological test for patients and carers

As eligibility criteria, patients and/or carers had to pass a basic technological test assessing home connectivity and patients and/or carers’ competences with the use of technology. The test consisted on the following 3 items:

1. Do you or your caregiver have an Internet connection?

- Yes
- No

1. Are you confident using:

- Smartphone
- Tablet computer
- Personal computer
- None of the above

1. Is your main carer confident using:

- Smartphone
- Tablet computer
- Personal computer
- None of the above

[This short test was considered as successfully passed if the patient and/or caregiver reported having an internet connection and being confident in the utilization of at least one of the proposed devices]

# The Integrated Care model

The Integrated Care (IC) model was based on a 5-step strategy: Case Identification, to select patients eligible for entering the program; Case Evaluation, to stratify patients based on clinical, environmental and social risk assessments; Work-plan definition, to plan personalized proactive and preventive interventions; Work-plan execution, to continuously monitor the evolution of the personalized care plan (through questionnaires, measurements with medical devices, educative material, and bidirectional communication wrapped-up in a patient self-management App); and, Discharge, to evaluate the overall intervention. The five steps were accommodated into a Smart Adaptive Case Management (SACM) web-based platform accessible to all involved professionals in the different settings (family physicians, hospital specialists and social workers), that guided professionals while keeping record of all relevant data. A detailed description of each of these steps as well as the technological tools that were used is presented below.

1. Case Identification

Potential patients to be included in the program were identified, based on electronic medical records (EMR) data. A nurse case manager contacted candidates before their discharge of an unanticipated admission to the hospital through the emergency room (ER). The case manager duties included the presentation of the study to the candidates, the collection of the patient’s informed consent form, the generation of a patient’s profile in the SACM platform, and the assessment of inclusion/exclusion criteria using such platform. Figure S1 shows the main screen of the SACM for the case manager. Figure S2 shows how the platform guides the case manager through the required steps for patient inclusion. At the time of new case generation, the case manager assigned the multidisciplinary team of professionals that would collaborate in the management of the patient, which could then be modified at any time. This first contact with the patient was usually performed in the patient’s hospital room using a tablet computer, which allowed for the required flexibility.


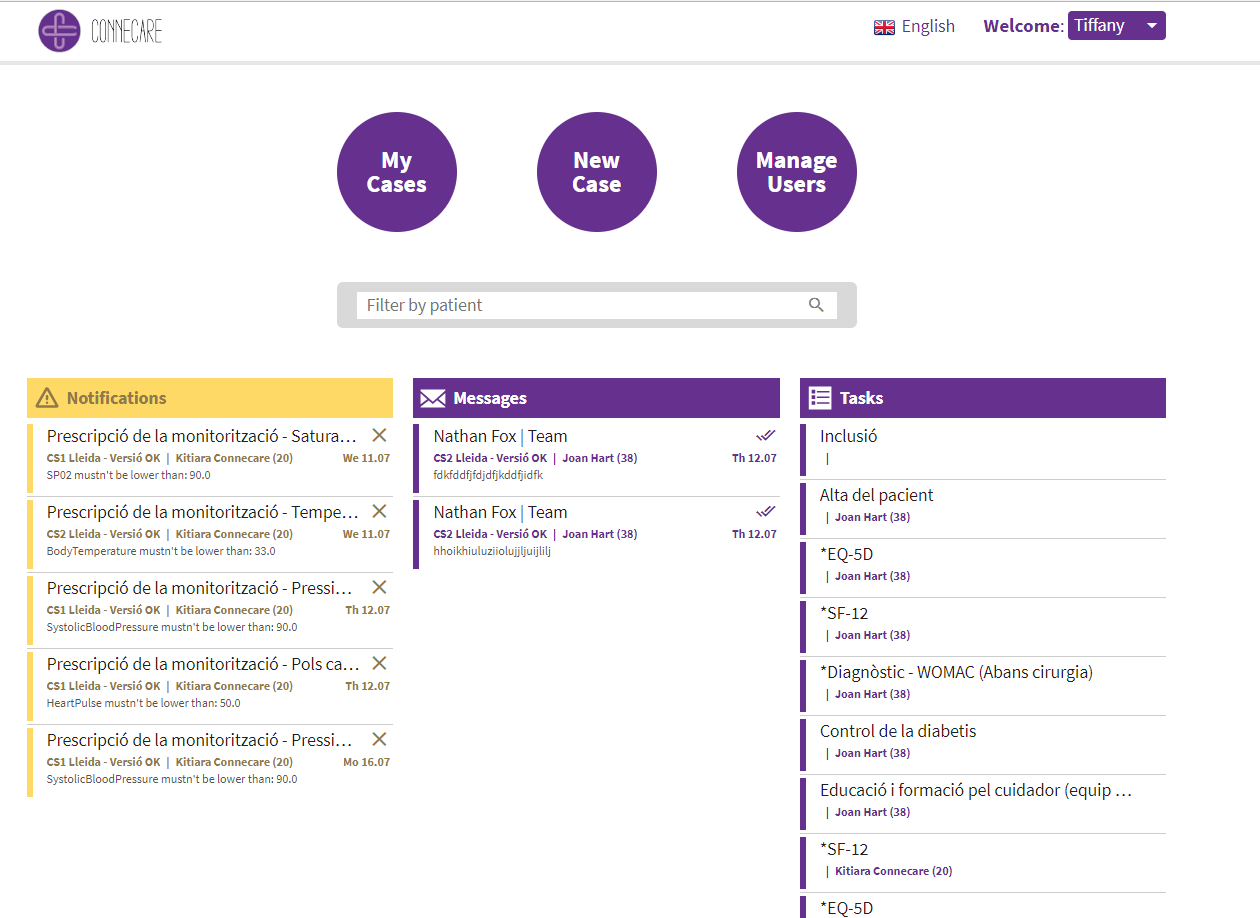


**Figure S1. Sample screenshot of the main screen of the SACM platform for the Case manager.**

**
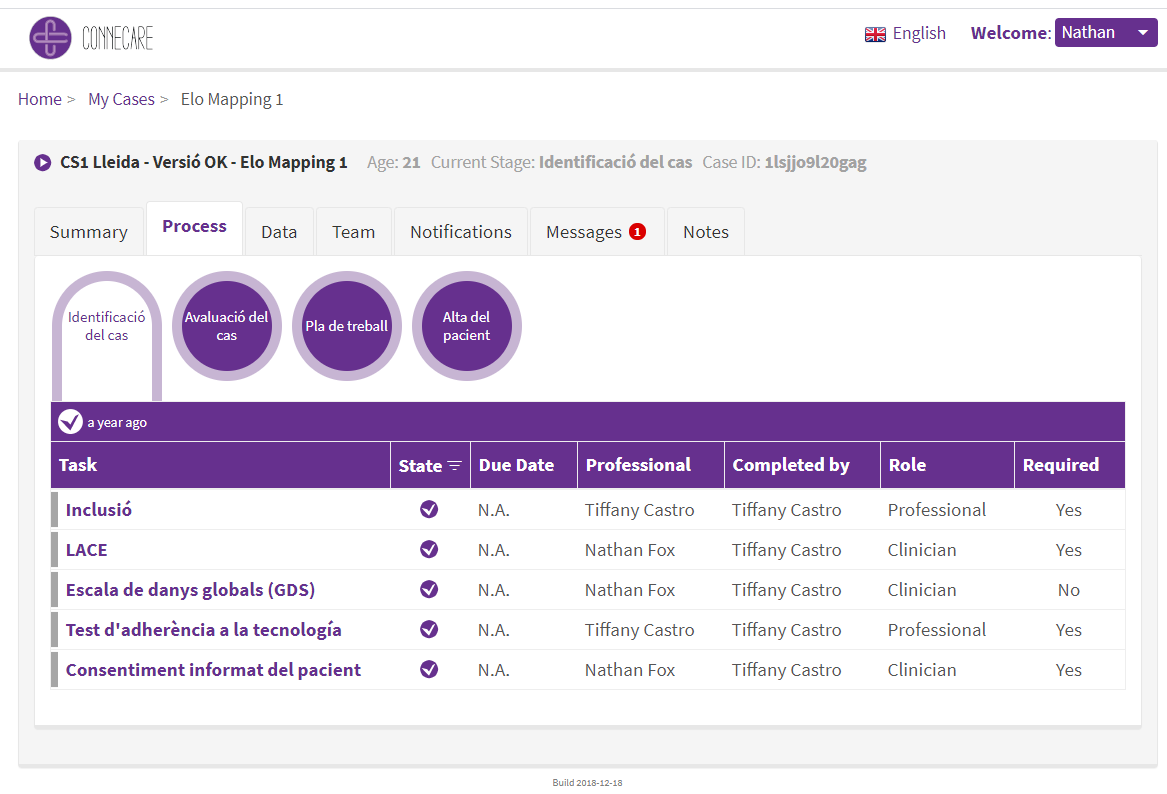
**

**Figure S2. Sample screenshot of the SACM platform, showing how the platform guides the case-manager through each step of case the identification.**

2. Case Evaluation

Once a patient was successfully included in the system, the first step consisted in assessing its baseline characteristics and performing the patient risk stratification based on clinical, environmental and social risk assessments. To this end, a set of questionnaires and tools implemented into the SACM were used (Table S2). This included tools assessing the characteristics of the patient and his environment: Functional Status and Autonomy (Barthel index for Activities of Daily Living); Comorbidities (Charlson index of comorbidities); Quality of life (SF-12); fall risk (Downton Fall Risk Index); mood (Hospital Anxiety and Depression (HAD) scale); mental status (Pfeiffer mental status questionnaire); nutritional status (body mass index); questions on communication and vision, characteristics of the patient’s dwelling, use of drugs, sleep habits, and use of tobacco and alcohol; and previous use of health resources. These assessments were performed by the nurse case-manager using a tablet computer. Once the information was collected (figure S3), the SACM automatically generated a summary screen for the patient (figure S4).


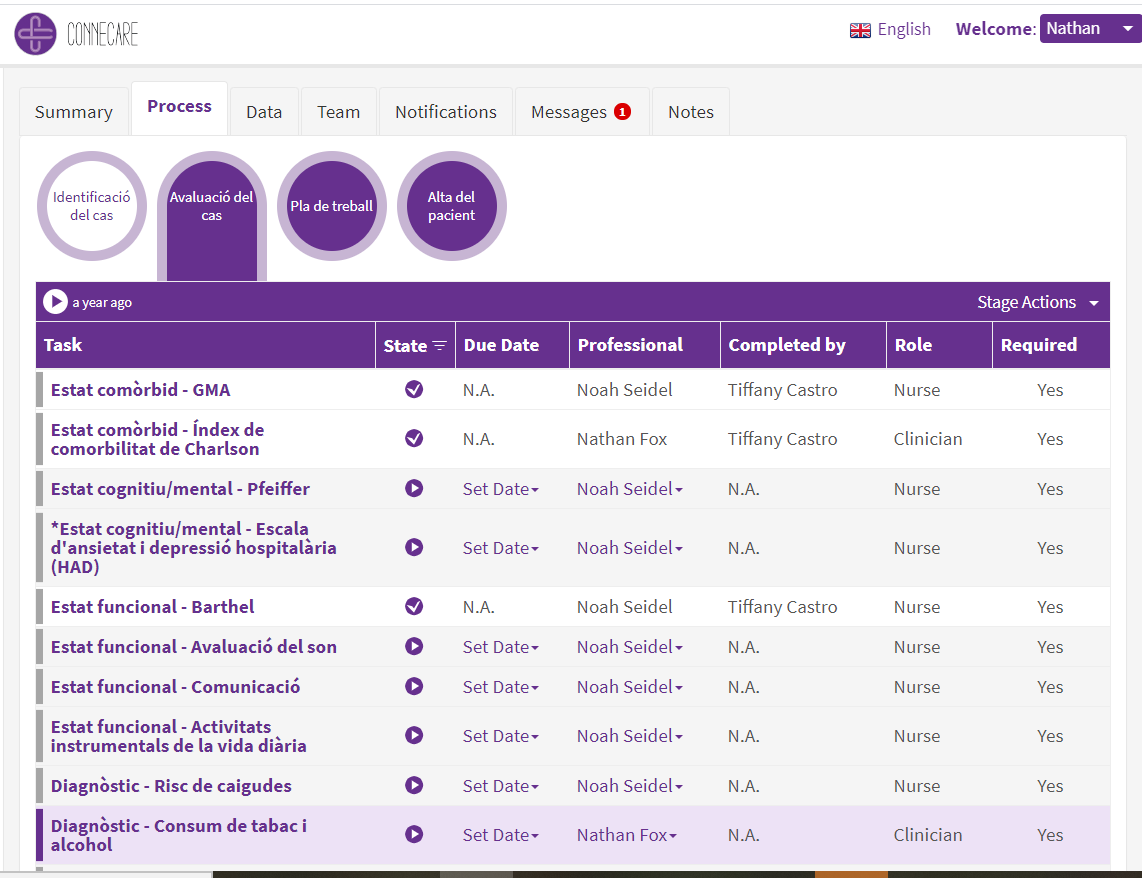


**Figure S3. Sample screenshot of the case evaluation process in the SACM platform.**


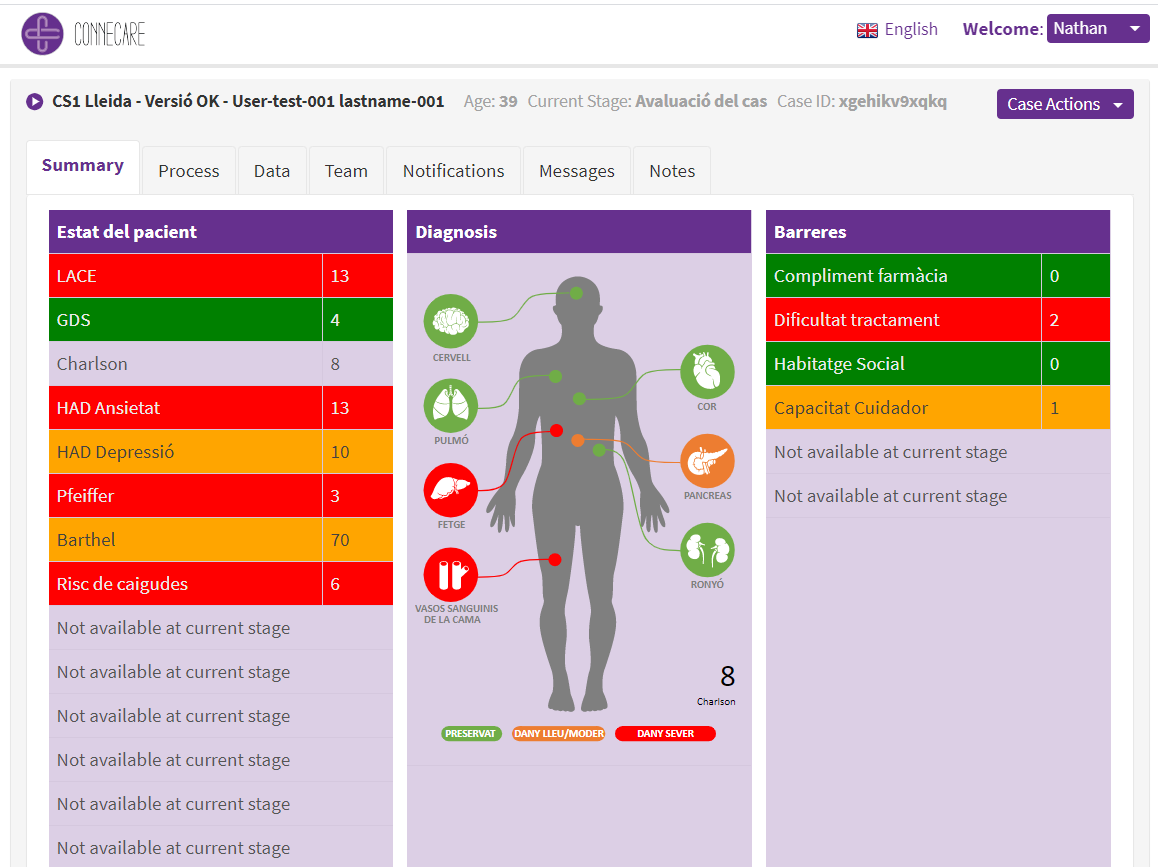


**Figure S4. Sample screenshot of the patient summary screen in the SACM platform.**

3. Work-plan definition

After the case evaluation, the case manager contacted the hospital physician who would define the main therapeutic strategies for the patient in agreement with the primary care team, which manages the patient in a day-to-day basis, and with the participation of the patient. A set of personalized proactive and preventive interventions would be agreed by all actors, and feed into the SACM platform. These included: a self-management app, with status and performance reports, a virtual coach with customizable automated feedback, full communication with the care team, and educational resources; a Fitbit Flex 2 digital activity tracker and any additional sensor deemed necessary by the care team (digital pulse-oximeter; digital scale; and, digital blood pressure (BP) monitor), all of them fully integrated into the self-management app; and any required drug treatment deemed necessary by the care team. Figure S5 shows sample screenshots showing some of the functionalities of the self-management app.


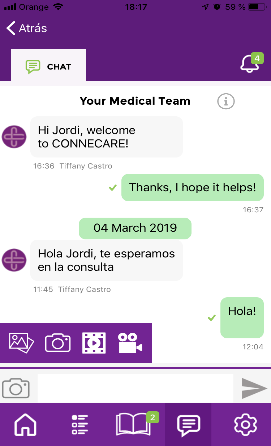

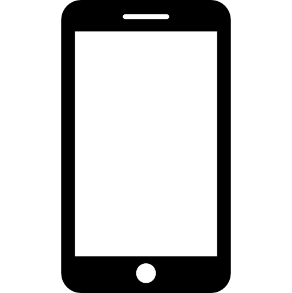

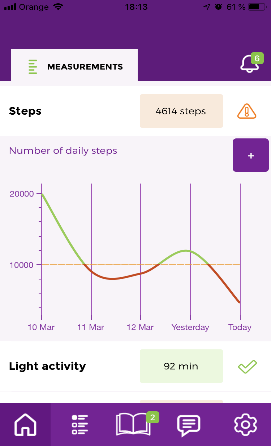

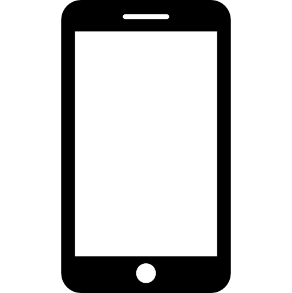

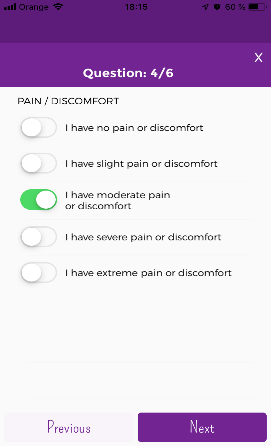

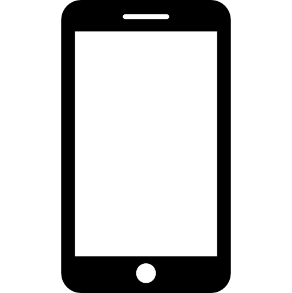

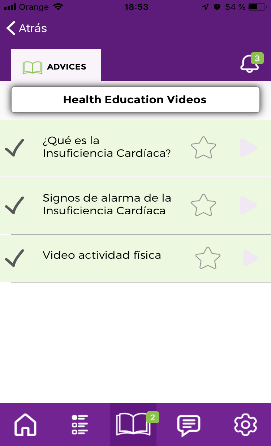

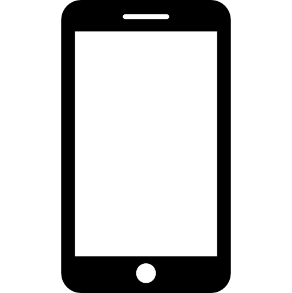


**Figure S5. Sample screenshots of the patient’s self-management app showing the administration of questionnaires, feedback on physical activity, communications with the care team, and educational resources.**

4. Work-plan execution

Once the work-plan was stablished, the progress of the patient was monitored throughout the work-plan execution phase. In this phase, the evolution of the personalized care plan was continuously monitored (through questionnaires, measurements with medical devices, educative material, and bidirectional communication wrapped-up in a patient self-management App). The patient’s self-management app, together with prescribed monitoring devices, was the key tool for self-monitoring the progress in the prescribed tasks as well as communicating with the care team (figure S5). The SACM was accessed by the members of the care team to check on the evolution of the patient and modify the therapeutic goals and/or tasks assigned to the patient. Moreover, the case manager overviewed the whole process and managed any alarms or signs, communicating with the patient and/or other members of the care team when needed. To this end, several summary screens were available to the case-manager in order to facilitate the simultaneous monitoring of all enrolled patients under its supervision (Figure S1 and S6). Among them, the geographical representation of patients in a map (mapping), combined with a traffic light system and multiple filtering and sorting options was very welcomed by participating professionals (Figure S6).


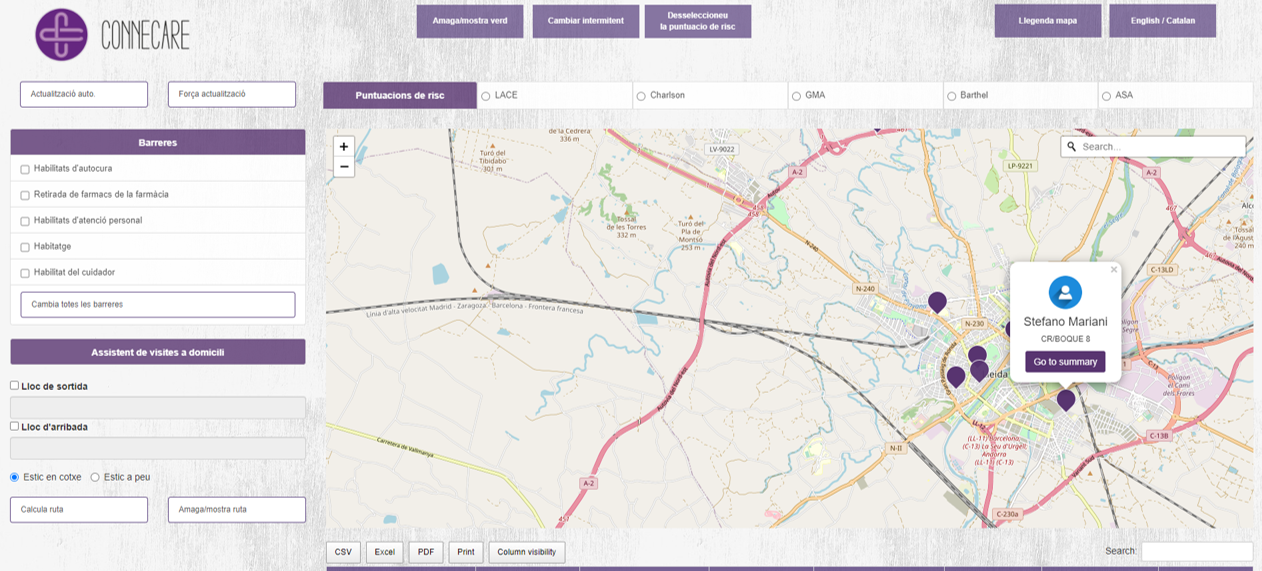


**Figure S6. Sample screenshot of the mapping functionally within the SACM.**

5. Discharge

The last step of the patient’s journey through the IC intervention was the discharge from the program, where the intervention was evaluated and all required feedback was collected. This task was performed by the case-manager and the hospital physician in care of the patient. It consisted on a visit where the patient and the professionals exposed their thoughts on the concluded IC program, the goals achieved, and the transfer of the management to the standard primary care management. The visit included the administration of several questionnaires and the collection of data from the EMR (Table S2).

# Technical structure of the supporting eHealth platform

The CONNECARE eHealth platform is a federation of subsystems each devoted to provide a set of goal-oriented functionalities, whose main components are the SMS app and the web-based SACM. Based on the concept of micro-services, the SMS provides intelligent tools to monitor patients (i.e., physical activity, sleeping, health status, drug adherence, simple rehabilitation tasks, and self-checked questionnaires) and to autonomously interact with them through engagement, rewards, and warnings through a recommender system. The SACM has extended functionalities for case modelling and execution, specifically tailored to the healthcare domain. Additionally, the SACM includes a Decision Support System to show patients in a map and to create routes for better organizing visits. The SMS and SACM interact each other through the CONNECARE Queue Manager, which connects both subsystems, orchestrates their communication, and provides an integration framework to link CONNECARE services to specific Electronic Health Records (EHR) and regional Personal Health Folders (PHF). Figure S7 sketches the architecture of the final CONNECARE system.


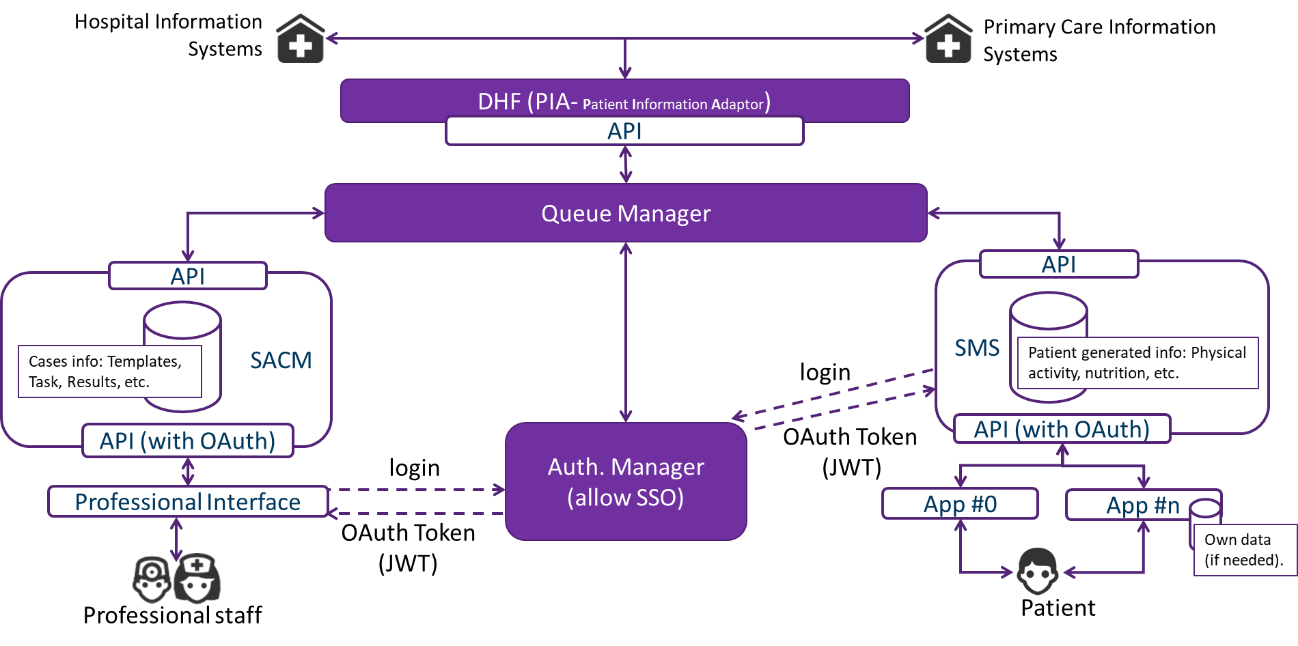


**Figure S7. Architecture of the CONNECARE eHealth system, including the web-based Smart Adaptive Case Management system (SACM) and the Self-Management System app (SMS).**

# Implementation efforts

On the one hand, the implementation of the IC model in Lleida required, first, the adaptation of the SACM platform. The self-management app needed to be fitted to the requirements and needs of the elderly CCP patients in Lleida. The SACM platform, needed to be the hub were all the professionals from different settings involved in the management of a given patient could exchange information, agree on the best management plan for each patient, and take specific actions in terms of treatment, monitoring and reactions when needed. Among others, the adaptations included the translation into Catalan and Spanish of all the self-management app and SACM platform contents, as well as a preliminary partial integration with legacy EMR in each setting (Argos SAP® (www.sap.com) and ECAP [1]). On the other hand, the implementation of the IC model required engaging a broad range of professionals and providing them with training and a fully functional access to the IC platform. Moreover, this required the emergence of new roles in the organization of the involved services. The already existing hospital case managers (that used to track a few proportion of patients), needed to be reinforced with a specific case-manager that took the role of both introducing the patient to the IC platform, and following-up the monitoring of the patients done by the involved health professionals.

# Implementation strategies and framework

The CONNECARE IC model was built upon the experience of on-going large-scale deployment programs in each of the participating sites, and the involvement of the main stakeholders in the process (staff and patients). A collaborative setting was established in order to capture the feedback of all actors of the integrated care process. The first phase of the development plan was the Co-design phase (Oct 2016 – Sept 2017), aiming at: i) adjusting the details of the service workflows to the characteristics of each site before initiation of the clinical studies; ii) participating in the definition of the characteristics of the model and supporting systems by defining the functional and non-functional requirements; and, iii) assessing suitability and acceptance of key indicators to be used for evaluation of the clinical studies. Next, the Refinement and Fine-tuning phase (Oct 2017 to Sept 2019) aimed to tests the model and involved larger groups of patients and staff. Unlike the Co-design phase, PDSA Cycles in the Refinement and Fine-tuning phase included the testing of an already operative system, even if some of its features were not fully developed until late stages of the project. This key difference required the implementation of actions like collecting feedback from real end-users.

CONNECARE followed a co-design approach using iterative 6-month Plan, Do, Study, Act (PDSA) cycles [2] to generate the design for the three integrated care services. The cycles consisted of a systematic series of steps for gaining valuable learning and knowledge for the continual improvement of the IC model and supporting technology. The main driving features of the PDSA framework were: (i) use of iterative cycles; (ii) test of change; (iii) small-scale testing; (iv) use of data over time; and, (v) appropriate documentation. Periodic reports on the activities, results and obtained feedback were elaborated and shared among key stakeholders. PDSA methodology provided overview; ownership; and, involvement of stakeholders who at all times had insight on the intervention process, while it encouraged management responsibilities to ensure focus, pace and self-discipline in the process. Moreover, the pragmatic nature of PDSA provided flexibility to develop interventions according to stakeholder’s feedback ensuring fit-for-purpose solutions, while providing the opportunity to build evidence for change and engage stakeholders as confidence in the intervention increased. Overall, PDSA was a successful approach for the development and implementation of the CONNECARE IC model.

Finally, the Consolidated Framework for Implementation Research (CFIR) [3] was used as part of the implementation assessment. The exercise provided a comprehensive view of the characteristics of the implementation process and allowed for the identification of facilitators/barriers encountered in the deployment process. Table S4 shows the assessment of the different CFIR constructs.

# References

1. ECAP. Catalan Health Dept. [http://salutweb.gencat.cat/ca/ambits_actuacio/linies_dactuacio
/tecnologies_informacio_i_comunicacio/ecap/](http://salutweb.gencat.cat/ca/ambits_actuacio/linies_dactuacio/tecnologies_informacio_i_comunicacio/ecap/) (last accessed 25/08/2020).

2. ACT academy. Plan, Do, Study, Act (PDSA) cycles and the model for improvement. NHS improvement 2018. <https://improvement.nhs.uk/documents/2142/plan-do-study-act.pdf> (last accessed 25/08/2020).

3. Consolidated Framework for Implementation Research (CFIR). <https://cfirguide.org/constructs/> (last accessed 25/08/2020).

# Supplementary tables

| **Table S1. Person-centeredness and continuity of care of patients in the Usual care and Integrated Care arms, according to use case.** | | | | | | | | | |
| --- | --- | --- | --- | --- | --- | --- | --- | --- | --- |
|  | All | | | Use case 1 | | | Use case 2 | | |
|  | UC | IC |  | UC | IC |  | UC | IC |  |
| **P3CEQ questions (% answering “always")** | (n=58) | (n=77) | *P* ^a^ | (n=28) | (n=48) | *P* ^a^ | (n=30) | (n=29) | *P* ^a^ |
| Did you discuss what was most important for YOU in managing your own health and wellbeing? | 42 (72%) | 53 (69%) | .652 | 23 (82%) | 35 (73%) | .361 | 19 (63%) | 18 (62%) | .920 |
| Were you involved as much as you wanted to be in decisions about your care? | 42 (72%) | 52 (68%) | .542 | 23 (82%) | 35 (73%) | .361 | 19 (63%) | 17 (59%) | .711 |
| Were you considered as a ‘whole person’ rather than just a disease/condition in relation to your care? | 47 (81%) | 64 (83%) | .754 | 24 (86%) | 41 (85%) | .972 | 23 (77%) | 23 (79%) | .807 |
| Did your care-team / providers involve your family / friends / carers as much as you wanted them to be in decisions about your care? | 43 (74%) | 53 (69%) | .501 | 23 (82%) | 35 (73%) | .361 | 20 (67%) | 18 (62%) | .712 |
| Have you had enough support from your care team / providers to help YOU to manage your own health and wellbeing? | 44 (76%) | 60 (78%) | .778 | 22 (79%) | 40 (83%) | .605 | 22 (73%) | 20 (69%) | .711 |
| To what extent do you receive useful information at the time you need it to help you manage your health and wellbeing? | 48 (87%) | 58 (89%) | .739 | 25 (89%) | 35 (90%) | .952 | 23 (85%) | 23 (89%) | .725 |
| **P3CEQ total score: mean (SD)** | 16.1 (3.3) | 16.3 (2.4) | .769 | 16.6 (3.0) | 16.4 (2.3) | .714 | 15.6 (3.6) | 16.0 (2.4) | .647 |
| ^a^ Chi2 test or T test as appropriate. Excluding patients answering DK/NA.  UC: Usual care; IC: Integrated care; P3CEQ: Person-centered coordinated care experience questionnaire; NCQ: Nijmegen Continuity Questionnaire. | | | | | | | | | |

| **Table S2. Continuity of care of patients in the Usual care and Integrated care arms, according to use case.** | | | | | | | | | |
| --- | --- | --- | --- | --- | --- | --- | --- | --- | --- |
|  | All | | | Use case 1 | | | Use case 2 | | |
|  | UC | IC |  | UC | IC |  | UC | IC |  |
| **NCQ G1-G5 (% "Agree" or "Strongly agree")** | (n=58) | (n=77) | *P* ^a^ | (n=28) | (n=48) | *P* ^a^ | (n=30) | (n=29) | *P* ^a^ |
| My care providers transfer information well to one-another | 17 (85%) | 32 (80%) | .637 | 12 (92%) | 24 (86%) | .548 | 5 (71%) | 8 (67%) | .829 |
| My care providers work together very well | 17 (81%) | 29 (76%) | .681 | 12 (92%) | 22 (85%) | .498 | 5 (63%) | 7 (58%) | .852 |
| My care providers are very well connected | 16 (84%) | 28 (78%) | .571 | 11 (92%) | 21 (88%) | .708 | 5 (71%) | 7 (58%) | .568 |
| My care providers always know what one-another is doing | 16 (80%) | 27 (79%) | .959 | 11 (92%) | 20 (83%) | .496 | 5 (63%) | 7 (70%) | .737 |
| I have to wait too long to obtain an appointment | 39 (67%) | 56 (76%) | .284 | 21 (75%) | 34 (72%) | .801 | 18 (60%) | 22 (82%) | .077 |
| **NCQ total G1-G5 score: mean (SD)** | 3.7 (0.9) | 4.0 (1.0) | .038 | 3.8 (0.7) | 4.1 (0.9) | .214 | 3.6 (1.0) | 4.0 (1.0) | .142 |
| ^a^ Chi2 test or T test as appropriate. Excluding patients answering DK/NA.  UC: Usual care; IC: Integrated care; P3CEQ: Person-centered coordinated care experience questionnaire; NCQ: Nijmegen Continuity Questionnaire. | | | | | | | | | |

**Table S3. Summary of the measurement tools used during the study.**

| **Table 4S. Description of the implementation strategy using the CFIR^a^ framework.** | |
| --- | --- |
| **Intervention characteristics** | |
| Intervention Source | Internally developed within the frame of the European H2020 CONNECARE project. |
| Evidence Strength & Quality | Moderate. No internal research was available and intervention effectiveness and cost-effectiveness were assessed along the implementation process. |
| Relative Advantage | Patients & caregivers: avoidance of unneeded travels to hospital or primary care centers; communication features; personalized feedback; monitoring; patient-centeredness; continuity of care.  Hospital professionals: organizational & clinical results; collaboration with primary care teams; management of groups of patients at a glance; enhanced monitoring features including alarms.  Primary care professionals: organizational & clinical results; collaboration with hospital professionals; continuity of care; management of groups of patients at a glance; enhanced monitoring features including alarms.  Social care professionals: organizational & clinical results; collaboration with clinical teams.  Managers: health outcomes; cost containment. |
| Adaptability | The CONNECARE eHealth-enabled IC model was developed to have a common inner structure that would then be tailored for each site and use case. Therefore, the system is highly adaptable by design. |
| Trialability | The implementation was progressive. Departing from the test of mock-components of the system, the final components were developed and fine-tuned using iterative 6-month cycles allowing continuously improving the system and undoing any troublesome feature. |
| Complexity | High complexity process requiring the re-elaboration of clinical protocols; redefinition of tasks & roles; technological deployment and support; professionals’ training; information and education for patients and carers; coordination among professionals in different settings; analysis of reimbursement modalities; testing of a system under development. |
| Design Quality & Packaging | Self-management app for patients and/or carers: freely available in Android and iOS official stores; functional design from day one.  SACM web-based platform: platform under constant development and fine-tuning, with addition of new features along the project; focus on functionality over front-end design until late stages of the implementation. |
| Cost | Implementation costs were covered by the CONNECARE project. Potential for cost-effectiveness was assessed during the project. Health-value generation was proven by the end of the project. |
| **OUTER SETTING** | |
| Patients’ needs & resources | Appropriate educational tools and training facilitated to patients and carers; patient-centered approach, with customizable feedback; patient choices & barriers taken into account and solved; enhancement of continuity of care; patients’ accessibility, satisfaction & opinions considered and taken as inputs for improvement; lean strategy minimizing costs. |
| Cosmopolitanism | The IC model was build-up as a functional integration of different healthcare providers at regional level. Part of the Catalonia region, rated as 4 stars reference site by EIP on AHA. |
| Peer pressure | Support from the single-public payer and internal managers. Expansion beyond Lleida Health-region limited by reimbursement modalities to other health-district provider organizations. |
| External Policy & Incentives | The European H2020 program provided funds for system development. The implementation was an internal decision within the Health region but had weak external support. The program should be considered as a learning experience. |
| **INNER SETTING** | |
| Structure Characteristics | Lleida Health region and its healthcare providers (hospitals, primary care and social services) had small-scale ongoing collaboration programs with very limited funds. Nevertheless, such collaborations allowed for a successful integration of the organizations within the project’s time, facilitating implementation and setting the basis for a potentially permanent deployment of the IC intervention. Key drivers for success were the leading role of professionals in the management (focus on efficiency); institutional aim for continuity of care; and, willingness for digital transformation. The vertical organization in clinical institutes was a relative barrier. |
| Networks & Communications | High professional engagement across settings. Enhanced communication features within the IC platform. |
| Culture | Mix of type 1 (team culture) and type 3 (entrepreneurial culture) with high engagement of professionals. |
| Implementation Climate | Positive climate for entrepreneurship, with high engagement of professionals in the aim of improving patient care. Direct participation of patients and professionals in the development and fine-tuning of the IC model and supporting systems. Initiative aligned with regional values and rules. No incentives or rewards were planned, but the role of all participants was acknowledged. |
| Readiness for implementation | Non-complete readiness for implementation at regional level, limited by fund availability, lacking complete integration with legacy electronic medical records. Good engagement of the leaders (champion-driven initiative). |
| **CHARACTERISTICS OF INDIVIDUALS** | |
| Knowledge & Believes about the intervention | Sustained positive perception of the intervention by both patients and professionals. Acknowledgement of enhanced health outcomes and positive impact on costs. |
| Self-efficacy | The CONNECARE IC model is designed to generate novel interactions between patients and/or professionals and foster self-efficacy. |
| Individual stage of change | Progressive achievement of active engagement of stakeholders throughout the development and fine-tuning phases. The more mature the system is the higher the engagement. |
| Individual identification with organization | The individual identification within each of the involved organizations was complement by the perception of the relevance of achieving continuity of care and being part of a broader cross-setting system within the health region. This was a facilitator for the implementation. |
| Other personal attributes | Tolerance, motivation, innovativeness, and learning style have been reinforced during the implementation process to cope with the handicap of using a system under development. |
| **PROCESS** | |
| Planning | The development and deployment of the CONNECARE eHealth-enabled IC model was planned and executed according to the Plan Do Study Act (PDSA) methodology ^b^, using 6-month iterative cycles. The number of involved patients and professionals increased throughout the project, thus having a gradual deployment that benefitted from the feedback received from professionals and patients. |
| Engaging | Implementation leaders (champions) triggered and conducted deployment with support, and direct interactions, with local managers. A specifically trained group of professionals with high degree of commitment and a transversal multidisciplinary approach contributed to consolidation. |
| Executing | The deployment plan was build based upon previous deployment experiences in Lleida and Catalonia. In the frame of the CONNECARE project, a detailed plan of milestones and deadlines ensured the achievement of results following a planned timeline. The utilization of iterative PDSA cycles for the development and fine-tuning of the IC model and supporting systems was very positive and allowed for valuable feedback from all stakeholders. PDSA methodology also allowed for the continuous monitoring of the implementation process. The strict periodicity of the cycles generated positive dynamics. Overall, the adoption of the PDSA methodology was a key facilitator element. |
| Reflecting & Evaluating | The use of the PDSA methodology allowed for a periodically assessment and evaluation of the development and implementation of the IC model and supporting systems. The results of each PDSA cycle were analyzed and included in a report, which was shared among key stakeholders. Cross-fertilization within other European sites participating in CONNECARE was an asset, as experiences were shared regularly. |
| ^a^ Consolidated Framework for Implementation Research (CFIR). <https://cfirguide.org/constructs/> (last accessed 25/08/2020).  ^b^ ACT academy. Plan, Do, Study, Act (PDSA) cycles and the model for improvement. NHS improvement 2018. <https://improvement.nhs.uk/documents/2142/plan-do-study-act.pdf> (last accessed 25/08/2020). | |
